# Exploring successes, barriers, and enablers in the one-year digital Healthy Weight Coaching

**DOI:** 10.1186/s12913-024-11876-2

**Published:** 2024-11-08

**Authors:** Anu Joki, Aila J. Ahola, Laura U. Suojanen, Kirsi H. Pietiläinen

**Affiliations:** 1 Healthy Weight Hub, Abdominal Centre, Helsinki University Hospital and University of Helsinki, Helsinki, 00290 Finland; 2 Obesity Research Unit, Research Program for Clinical and Molecular Metabolism, Faculty of Medicine, University of Helsinki, Biomedicum 1, Haartmaninkatu 8, PO Box 63, Helsinki, 00014 Finland

**Keywords:** Weight management, Digital coaching, Healthcare professionals, Lifestyle change intervention, Qualitative interview study, Success factors, Self-compassion, Internal motivation, Holistic well-being, Individualized support

## Abstract

**Introduction:**

Healthcare professionals’ perspectives are often overlooked in the evaluation of digital weight loss interventions. Thus, we examined how healthcare professionals perceive patient success in a one-year web-based weight management program, the Healthy Weight Coaching, aiming to identify key success factors and common challenges within the coaching process.

**Methods:**

Thematic analysis was conducted on ten semi-structured interviews with healthcare professionals from the Healthy Weight Coaching. Interviewees included individuals of both sexes, with an average age of 41 years, ranging from 10 months to 5 years of coaching experience, and treating 90 to 500 patients.

**Results:**

Three main themes emerged from the analysis: (1) Positive progress during the Healthy Weight Coaching, (2) Factors facilitating success, and (3) Barriers hindering progress. The coaches noted significant positive changes among patients, including increased self-compassion, reduced weight-related stress, and a shift toward holistic well-being. Improvements in eating habits, physical activity, and vegetable consumption were observed, along with reductions in binge eating behaviors. Personal factors such as internal motivation and engagement with the program were crucial for success. Additionally, aspects of the Healthy Weight Coaching program, such as its individualized and supportive environment, flexible coaching approach, and the pivotal role of coaches, were seen as facilitators of success. Patients valued being treated equally and acceptingly and fostering trust and collaboration. However, challenges such as burdensome life situations, limited resources, and inadequate support posed significant barriers to progress. Unrealistic goals and societal pressures were also observed to hinder successful weight management efforts.

**Conclusion:**

A comprehensive, individualized approach addressing resource limitations and societal norms can enhance long-term success in weight management programs like the Healthy Weight Coaching, ultimately promoting overall well-being.

**Supplementary Information:**

The online version contains supplementary material available at 10.1186/s12913-024-11876-2.

## Introduction

Obesity is a widespread global health concern, with its prevalence steadily rising in recent decades [1]. Despite significant efforts, traditional weight management approaches often yield limited long-term effectiveness [2]. However, digital lifestyle change interventions are increasingly recognized as promising tools for fostering sustainable weight loss and overall well-being [3]. These interventions, acknowledged for their scalability and accessibility, offer personalized support to individuals striving for healthier lifestyles [4, 5].

At the same time, the increased use of digital devices and social media may introduce new challenges. Modern lifestyles, characterized by high smartphone use and social media engagement, have been linked to adverse health behaviors. Social media addiction, for example, is associated with increased sedentary time, poor sleep quality, and lower physical activity levels, all of which can negatively affect weight management efforts [6, 7]. Additionally, excessive digital engagement may contribute to psychological issues such as anxiety and depression and strain social relationships.

Despite these challenges, the digital landscape provides a unique avenue for delivering tailored interventions. By combining behavior modification, education, social support, and self-monitoring, digital programs empower participants to make healthier choices [8]. Numerous randomized controlled trials and systematic reviews have consistently demonstrated the efficacy of digital interventions in promoting weight loss and improving health outcomes [8–10]. For example, Zheng et al. [10] observed in their systematic review and meta-analysis that holistic mHealth interventions, utilizing mobile devices such as smartphones and tablets for healthcare purposes, were significantly associated with weight loss and reduced perceived stress levels, further highlighting their potential efficacy in promoting health outcomes.

Moreover, recent research emphasizes the clinical benefits of digital interventions for specific populations. In a single-arm, three-site clinical trial, a scalable, virtually delivered weight management program tailored for individuals with type 2 diabetes showed clinically meaningful effects on various health markers [11]. Participants experienced significant improvements in glycemic control, body weight, and psychosocial outcomes, alongside enhancements in quality of life. Furthermore, a recent randomized controlled trial found that higher engagement with digital weight loss intervention correlated with greater weight loss, which was facilitated by personalized feedback that offered individual progress updates and strategies to overcome barriers [12].

Despite extensive research on the efficacy of digital interventions in promoting health, there remains a gap in understanding individual experiences of success, both from the perspective of participants and the healthcare professionals who deliver these interventions. This qualitative study aimed to address this gap by exploring the perceptions of success among patients engaged in a digital lifestyle change intervention, as articulated by healthcare professionals serving as personal coaches in the Healthy Weight Coaching program. These professionals play a crucial role in guiding and supporting individuals on their health journey, offering firsthand insights into the multifaceted dynamics of success within digital interventions for weight management. This study focused on capturing the healthcare professionals’ perspectives by exploring the following questions: According to healthcare professionals, (1) what are the individual success experiences in the Healthy Weight Coaching program? and (2) what factors support or hinder individuals in achieving their weight management goals?

## Methods

We conducted ten interviews with healthcare professionals employed at the Healthy Weight Coaching. The Healthy Weight Coaching is a 12-month interactive digital program offered by the Abdominal Center of the Helsinki and Uusimaa Hospital District through the web-based platform HealthyWeightHub.fi [13]. HealthyWeightHub.fi is part of the Health Village (Terveyskyla.fi), which is a digital portal coordinated by Helsinki University Hospital and developed in collaboration with five university hospital districts and several patient associations in Finland [14].

The real-life weight management program, the Healthy Weight Coaching, provides comprehensive support for individuals with overweight or obesity. It is a referral-based, nationwide digital program for adults with a BMI ≥ 25 kg/m² [15]. The program includes 52 automated weekly training sessions covering diet, physical activity, sleep, psychology, and overall health, with the flexibility to complete at one’s own pace. A healthcare professional acts as a personal coach, providing tailored support through one-on-one digital communication. On average, patients engage with their coaches 40 times, and optional group chats offer peer support. The program is actively updated to ensure that patients receive the most current and evidence-based guidance [16].

To secure the anonymity of the interviewees, the demographics are given in a general way. Both women and men were interviewed, and no other gender identities were reported. The mean age of the interviewees was 41 years (SD = 9.3). Interviewees represented various medical professionals, including nurses, nutritionists, and physiotherapists, who had worked several years in different positions in health care. The coaches’ work experience at the Healthy Weight Coaching ranged from 10 months to 5 years. The number of patients treated ranged from 90 to 500, reflecting differences in work hours, as some coaches worked full-time, while some worked part-time.

The interviewees were recruited through purposive sampling during a monthly coach meeting where the first author presented the study and invited interested coaches to contact her. Also, the invitation letter was posted to the coaches’ working platform in Teams. Participating in the research was voluntary, and all interviewees provided informed consent before the interviews. Data were collected between June and September 2023 through semi-structured theme interviews, which offer flexibility and depth to data collection [17]. The interview guide (Appendix 1) covered various topics related to the Healthy Weight Coaching, providing a structured framework while allowing for the exploration of interviewees’ perspectives [18]. However, the themes relevant to this study focused on exploring success experiences within the program. Specifically, the interview discussions centered on elucidating the understanding of success and identifying key factors contributing to or hindering success.

The coaches were encouraged to share success stories, thus providing valuable insights into exemplary outcomes within the program. Additionally, the interviews explored factors associated with unsuccessful situations or program discontinuation. Prior to the study, the interview guide was pilot tested with a representative from the target group to ensure clarity and relevance. The interviewer (AJ), who had a working relationship with the interviewees, had ample experience conducting interviews. Interviewees were explicitly instructed at the beginning of each interview to share their experiences without directly revealing identifiable information. However, if names or other identifiable details emerged during the interview, AJ replaced them during transcription to ensure the anonymity of individual coaches and patients in the results. The first author conducted all the interviews and transcriptions.

All the interviews were conducted in Finnish on a single occasion. They were carried out and recorded via Zoom over a secured connection provided by the University of Helsinki or, in one instance, via Teams secured by the Helsinki University Hospital. The interviews lasted an average 71 min, ranging from 47 to 95 min. The final data consisted of about 12 h of recordings. The saturation point, when new data ceases to provide additional insights or perspectives, was used to guide the data collection process, ensuring a comprehensive understanding of the topic was achieved [19].

All interview data were transcribed verbatim and analyzed using inductive thematic analysis [18]. The first author systematically read and re-read each transcript several times and then grouped text quotations containing relevant information to the research question into codes in a separate Word document. The coded texts were reviewed and summarized in the following phase to conceptualize the codes into meaningful subthemes and themes. Throughout the process, the researchers together discussed and evaluated the adequacy of coding, ensuring the accuracy and comprehensiveness of the analysis. Finally, as presented by Braun and Clarke [18], the essence of each theme was identified, and three main themes were formed. The final step of the analysis involved producing a report that included vivid quotations from interviews to validate the conducted analysis.

### Reflexivity statement

The first author (AJ), a postdoctoral researcher, specializes in weight management and lifestyle modifications, with extensive experience in qualitative research, including data collection, analysis, and manuscript writing. With an educational background in nutrition sciences, AJ’s interdisciplinary research integrates health sociology and psychology with nutrition and public health. AJ also brings practical insights from her experience as a weight management group instructor.

The second author (AJA) is an adjunct professor of Nutrition and Public Health and has extensive experience in both clinical and epidemiological research. Her studies have mainly focused on weight management and self-management of type 1 diabetes. AJA has extensive experience in writing protocols, collecting data, conducting analyses, and writing manuscripts. AJA’s academic education combines nutrition (major), psychology (minor), and public health (minor).

The third author (LUS) is a doctoral researcher, nutritionist, and licensed nurse employed as a development manager at the primary health care unit of HUS. Her research centers on eating behavior and obesity, areas to which she has devoted her professional career. Her work addresses eating disorders and obesity, representing a core aspect of her expertise.

The last author (KHP) is a Professor in Clinical Metabolism and a specialist in internal medicine. As the chief physician of the Healthy Weight Coaching, KHP oversees the program’s medical integrity and scientific accuracy. Her extensive research, including investigations into the effects of diet, surgical interventions, and medications on weight loss, as well as the metabolic impacts of obesity and type 2 diabetes, underscores her dedication to advancing scientific knowledge in these areas. Additionally, KHP supervises the coaches to ensure the Healthy Weight Coaching aligns with the current medical standards and practices.

## Results

In this study, three main themes emerged from the perspectives of the coaches who shared their insights of the individual success experiences based on their interactions with patients in the Healthy Weight Coaching program: (1) Positive progress during the Healthy Weight Coaching, (2) Factors facilitating success, and (3) Barriers hindering progress (Table 1). These themes encapsulate the diverse experiences observed by the coaches and highlight the factors contributing to or hindering success within the program. Each theme is examined in detail, along with the underlying sub-themes, to provide a comprehensive understanding of the dynamics influencing success as observed by the coaches.

**Table 1:** Overview of the main themes and sub-themes emerged from the analysis

Main themes	1. Positive progress during the Healthy Weight Coaching	2. Factors facilitating success	3. Barriers hindering progress
Sub-themes	1. Changes in the mental state	1. Personal Factors	1. Personal Factors
	2. Changes at the practical level	2. Features of the Healthy Weight Coaching	2. Features of the Healthy Weight Coaching
			3. Societal understanding

### Positive progress during the Healthy Weight Coaching

In our analysis, two subthemes emerged: *Changes in the mental state* and *Changes at the practical level*. These subthemes encompassed a range of changes observed and reported by the coaches in patients’ attitudes and behaviors over the year. Many patients experienced shifts in their life values, transitioning from prioritizing work and other external obligations to prioritizing well-being. The coaches noted significant changes in self-compassion, which was reflected in positive attitudes towards exercise, food, and self-image. Accomplishments such as discovering relaxation and flexibility in eating habits contributed to an enhanced self-perception.

One coach explained:

“*There’s this really strong sense of mercilessness and self-punishment*,* and somehow the thought of ‘I don’t deserve anything good’ comes up a lot. So*,* it’s encouraging to read the messages when someone starts to get out of that mindset. And now the thought is like*,* ‘I deserve all the good in the world’ and that I can prioritize rest and relaxation for my body without feeling the need to punish myself.*” (I2).

The coaches commonly reported a significant shift in the patients’ priorities towards holistic well-being, with lifestyle changes focusing on improving the quality of life rather than weight control or loss. According to the coaches, the key realizations included reduced emphasis on weight, resulting in decreased stress and weight having less influence over patients’ lives. This shift, as noted by the coaches, is often facilitated by the professionals’ careful guidance, helping patients reframe their goals and adopt a broader perspective on well-being.

The following quotations exemplify this:

“*The perspective on weight becomes more neutral: for some*,* they accept that weight is what it is*,* whereas initially*,* there’s significant negativity and shame regarding size*,* and weight acts as a barrier to living fully. However*,* when you read the feedback forms and notice that they have become gentler towards themselves*,* I think it’s also a great success.*” (I4).

“*In my opinion*,* the greatest successes often involve the shift away from solely focusing on the number on the scale towards a broader perspective on overall well-being and resilience. It’s quite common to witness this happening*,* where individuals gradually realize how they’re freeing themselves from the limits of their own thinking. Success has been defined by a specific number on the scale or weight loss*,* which might have been the only measure of their lifestyle changes up until that point. But gradually*,* over the year*,* there’s a shift in mindset. You start to read thoughts like*,* ‘Actually*,* weight isn’t the most important thing to me anymore; it’s my well-being.’ And with that realization comes a newfound flexibility in thinking.*” (I7).

The coaches also underscored that the Healthy Weight Coaching program helped patients move away from harmful dieting mentalities. Initially, patients categorized foods as ‘good’ or ‘bad,’ followed strict dietary guidelines, and experienced weight cycling. Nevertheless, the Healthy Weight Coaching program’s holistic approach, combined with the coaches’ supportive and individualized guidance, empowered patients to break free from these harmful patterns, including compensatory behaviors such as skipping meals or over-exercising. As a result, eating habits became more flexible and enjoyable, reducing self-blame.

“*One example is a woman in her early thirties. At the beginning of the coaching program*,* she had numerous food restriction lists and had adopted many strict rules from her childhood home. Then*,* she relaxed a lot more about eating*,* and somehow*,* she managed to change her mindset a lot*,* realizing that she could eat bananas and didn’t feel as much guilt anymore if she ate*,* for example*,* treats. I remember her emphasizing that it’s more relaxed now. She had found relaxation in eating…I can’t remember if she had lost a lot of weight by then*,* but still*,* there were many changes happening at that mental level.”* (I3).

Moreover, changes in patients’ attitudes became evident as they confronted the prevailing negative beliefs and misconceptions about exercise and dietary habits they had had before their involvement in the program. According to the coaches, many individuals believed exercise needed to meet specific duration and intensity requirements or that sweating was necessary for it to be beneficial. Additionally, exercise had often been viewed solely as a means to burn consumed calories. Negative self-perceptions, such as feeling unfit for exercise due to weight or fitness level, were common, as were feelings of exclusion from certain activities due to internalized labels. Overcoming these beliefs required considerable time and effort, but program participation along with the encouragement from the coach both facilitated this process, as illustrated in the following quotation:

“*When one succeeds in breaking free from the beliefs or labels that have built up over the years*,* such as the idea that I am inadequate and incapable*,* and even though others can participate in sports or other activities*,* I won’t due to some negative attribute*,* the unraveling of that bundle of beliefs becomes a significant factor.”* (I2).

Furthermore, the coaches emphasized that the patients reported increased autonomy and self-awareness. Their self-efficacy had improved, enabling them to tackle tasks they previously believed impossible. Additionally, they had taken greater responsibility for their lifestyle habits and initiated changes. Moreover, lifestyle change was viewed as an ongoing journey with the potential for continuous improvement.

“*It’s incredibly wonderful to read those messages where the increase in self-efficacy and improved self-understanding are reflected; my clients have told me that during the program*,* they have learned to believe in their ability to make changes and gained a better understanding of their own needs.”* (I4).

Now, we turn our attention to some behavioral changes observed within the sub-theme of *Changes at the practical level*. The coaches noted a decrease in binge eating among many patients who had previously faced challenges with eating management. One coach articulated this:

“*Many of my patients*,* and I suspect that nearly everyone in this group*,* initially have high BES (binge eating scale) scores. When they engage in this program and allow themselves to begin eating regularly*,* it helps to decrease the tendency for binge eating*,* and many of them manage to overcome it. In most cases*,* this improvement is also reflected in their weight.”* (I6).

Furthermore, other common changes mentioned by the coaches included adopting regular eating patterns, increasing vegetable consumption, and engaging in physical activity. These behaviors gradually became habitual during the program, initially requiring conscious effort but eventually becoming automatic parts of patients’ lives. The Healthy Weight Coaching participants expressed newfound joy in movement and exercise, emphasizing enjoyment rather than solely focusing on weight loss. These positive lifestyle modifications had led to various improvements, including weight loss, enhanced sleep, increased vitality, and improved coping at work. The patients also had reported reductions in waist circumference and the prevention of further weight gain as outcomes of the Healthy Weight Coaching program. Although not formally measured as part of the program, many patients had reported positive changes in laboratory measurements, such as blood glucose, cholesterol levels, and blood pressure.

*“One thing that people often mention is that there hasn’t been much change in weight. However*,* when they’ve undergone lab tests*,* their glucose levels haven’t been as high anymore*,* or their high cholesterol levels have decreased. These changes often become apparent at an early stage.”* (I8).

### Factors facilitating success

Two sub-themes, *Personal Factors* and *Features of the Healthy Weight Coaching*, emerged from the data, describing the factors contributing to the patients’ success throughout the program. All the coaches emphasized that having a harmonious life situation characterized by reduced stress and sufficient resources facilitates success in weight management. In modern life, high stress levels and busy schedules make it difficult for many patients to find balance. The program’s digital structure enabled patients to integrate the necessary lifestyle changes into their daily routines. The coaches also stressed the importance of a calm and supportive environment for initiating and sustaining lifestyle changes, which should also be taken into account when the patients are referred for treatment.

Moreover, the coaches emphasized that establishing and maintaining regular meal patterns was key to successful weight management. The ability to plan and prepare for challenges—often exacerbated by the fast-paced nature of modern living—was vital for success. The digital platform allowed patients to access information and tools for better planning, reducing impulsive decisions during stressful times, such as after a busy workday. The coaches described that through discussions with patients, they were able to support them in identifying their own strategies for managing these everyday challenges rather than providing direct solutions. This approach encouraged patients to develop their own plans and prepare for potential obstacles, making it easier to maintain healthy habits under pressure. They shared examples from the patients who had used planning as a strategy to avoid unfavorable situations, such as the following:

“*For example*,* when it comes to dinner*,* successful individuals plan ahead by preparing meals in advance with plenty of vegetables or a salad stored in the fridge. This is crucial*,* because when you get home hungry*,* it’s really tempting to swing by the drive-thru or grab a snack before dinner is ready*,* and then you end up eating something unhealthy. Conversely*,* those who have their dinner ready in advance tend to succeed quite well in this regard.*” (I4).

Support from social networks, including family, friends, and peers, was viewed as invaluable in maintaining motivation and adherence to healthy behaviors. When traditional social networks were less accessible, the program’s digital features provided an additional support, enabling patients to remain connected and engaged, even when isolated from their immediate social circles. Moreover, active engagement in both the change process and the program itself was seen as pivotal for successful weight loss and maintaining motivation. The coaches observed that active involvement in the program and willingness to share thoughts and feelings were crucial for positive outcomes. Additionally, motivation, especially internal motivation, was considered critical for initiating and sustaining lifestyle changes.

“*And the successes have specifically been with those individuals who have been motivated*,* they have familiarized themselves with this beforehand. For many*,* the process has already begun before they’ve even come to this program*,* for example*,* they have lost weight or there’s been some other trigger such as they have gotten some extra resources*,* I think in these cases the coaching program really supports the process very well.”* (I5).

*“I think it’s the whole package*,* the situation in your everyday life is auspicious*,* you have time to think about these topics and you are ready for it*,* and then you get sufficient support from your loved ones for the change when needed*,* so all those variables in your daily life undoubtedly contribute to promoting your well-being when you consider it as a whole.*” (I7).

Moreover, the ability to confront and overcome obstacles and to adapt to changing circumstances was highlighted as crucial for long-term success in weight management. These skills were observed to develop during the program, which is particularly significant in light of the increasing pressures of modern living, such as the hectic pace and rising stress levels, as well as the weakening of traditional social support networks like family and community ties. Patients had faced these obstacles, including social isolation and the demands of contemporary lifestyles, but were able to build resilience and maintain their progress:

“*One thing that came to my mind*,* from what one of the participants said*,* was that when facing setbacks - evident for all - what he had noticed was that when facing setbacks and experiencing negative emotions*,* they weren’t as intense as they had been before. And that can often be the case; when experiencing intense negative feelings*,* it can crush*,* slow down*,* or even prevent the continuation of change. So*,* resilience is needed*,* and it has improved. You can better face setbacks and bounce back from them*,* he articulated it so well.”* (I1).

Furthermore, the coaches discussed the role of medication in aiding success. They noted that in some cases, anti-obesity medication supported weight loss efforts, particularly when coupled with dietary modifications. They recounted that some individuals, upon starting medication, experienced reduced preoccupation with food or diminished cravings compared to their previous habits.

Another aspect contributing to success lies in the elements of the Healthy Weight Coaching program itself. Patients found the personalized and supportive atmosphere, combined with the program’s flexible digital approach, particularly beneficial. The program’s digital features, including online chat meetings and MealLogger groups, fostered a sense of community and facilitated the exchange of experiences among patients. Furthermore, the program’s digital accessibility and flexibility, which allowed individuals to engage with the program at their convenience, were seen as advantageous, promoting autonomy and adherence to the program.

Furthermore, the role of the coaches was deemed crucial – patients expressed gratitude for being able to share their lifestyle change journey with a supportive individual, a real person who was genuinely interested in their progress. Many patients, particularly those who felt lonely, greatly appreciated this aspect. As one coach (I6) described: *“Some say that just this question of how are you doing or sending an encouraging message can really make a difference in whether they continue with the coaching*,* so it can have some impact on people as well.”* The coaches mentioned that the patients found it motivating and fostering a sense of connection to know that there was always the same person available to offer support, encouragement, and celebrate successes. The coaches were seen as mirrors, reflecting on individuals’ progress and boosting their confidence.

“*Yeah*,* I somehow think that there’s also a certain kind of positive pressure that definitely comes with it when you know that someone*,* like your coach*,* is reading these texts and tracking your progress. It’s also something that many seem to want*,* a little nudge forward.”* (I3).

“*Some of the participants find it incredibly valuable that someone follows them throughout the year and is genuinely interested in their well-being and affairs.*” (I4).

Moreover, the coaches personalized the program to suit individual needs, tailoring coaching sessions and additional tasks accordingly. The coaches reported that the personalization process started with the referral team, where the most suitable coach was chosen based on the information provided about the patient’s circumstances in the referral. Also, during the initial phone call and through messages with patients, coaches gathered further details to refine their coaching approach. Additionally, offering supplementary materials, personalized messages, and addressing unique challenges enriched the sense of individuality and demonstrated a tailored service. One coach elaborated on her approach to creating this personalized experience:

“*I think one of the biggest things is that feeling of familiarity that comes to the participant*,* even though*,* of course*,* now that I have so many patients*,* I can’t remember every detail of their lives*,* but I do remember quite a lot and I make an effort to remember as much as I can by reading those previous conversations and texts*,* so I can catch up on where we are*,* and so that the patient gets the feeling that I remember their situation and I’m interested in how they are doing and how the coaching is progressing*,* so I definitely think that’s a crucial part*.” (I9).

Furthermore, the ideology of the Healthy Weight Coaching emphasizes a respectful and non-judgmental approach towards patients, creating a safe space where individuals feel accepted and understood. As one of the coaches (I6) explained: *“Someone even asked me if they should weigh themselves in front of someone else*,* and I said*,* ‘You don’t have to weigh yourself in front of anyone!’ People have all sorts of traumas*,* but they can participate openly and without fear here. They can make the changes they’re capable of*,* and no one comes in with this attitude of ‘you need to do more’.*” Patients appreciated being treated as equals and valued for who they are, free from judgment or stigma. This approach fosters a sense of respect and understanding rather than making individuals feel like failures for not achieving weight control. Unfortunately, many patients had shared with the coaches that such an approach was uncommon in obesity care, expressing pleasant surprise upon joining the program.

*“Yeah*,* some of the people I coach have mentioned that they’re even surprised by the level of compassion and sympathy they encounter. It’s been a positive surprise for some of them*,* how accepting this whole process can be.”* (I9).

The coaching program adopts a holistic approach, addressing not only diet and exercise but also psychological aspects such as self-reflection, coping strategies, and life circumstances. Central to its ideology is promoting self-examination and personal growth by prompting individuals to reflect on their habits and challenges. The year-long program provides extensive support for sustained behavior change and deeper exploration. Throughout the coaching journey, individuals are challenged to make meaningful, long-term changes in their lives.

One of the coaches exemplified this:

“*Well*,* I’ve come to the conclusion that the biggest thing is taking the time and pausing to really think about things. I hear a lot from those I coach that the biggest thing*,* although the concepts may be familiar*,* is actually having to stop and ponder*,* go through it all. Especially when life is busy and your well-being is maybe 18th on the list among everything else*,* this provides the opportunity and kind of kicks you into gear to take that time and go through them. And then*,* as that moment potentially repeats every week*,* one’s own project is quite nicely maintained*,* and one’s mind processes these things in between*,* too.”* (I8).

### Barriers hindering progress

Finally, three sub-themes described the Barriers hindering progress: *Personal Factors*,* Features of the Healthy Weight Coaching*, and *Societal understanding*. First, various personal, psychological, physical, and social factors significantly impacted the lifestyle change and weight management. According to the coaches’ experiences, burdensome life situations and limited resources, such as hectic schedules or stress, were crucial challenges in weight management.

*“I have patients who think*,* and perhaps rightly so*,* that they are in a desperate situation in life. They face numerous health challenges*,* mental health issues*,* loneliness*,* and poverty. Their life situation is miserable and narrow*,* making it impossible to even consider going to the grocery store to make healthy food choices or joining a gym and attending exercise classes. One of my coached individuals told me that on a good day*,* she listens to audiobooks in bed*,* and on a bad day*,* she doesn’t. When the starting point is like that*,* we cannot even begin discussing exercise.*” (I9).

Coaches noted that they had to modify their coaching methods to accommodate these life challenges, ensuring their approach remained relevant and supportive for each patient’s unique context and current capacity:

“*Yeah*,* and the exhaustion*,* there’s so much overwhelming stress and fatigue right now. People can’t sleep*,* stress levels are high*,* barely hanging on at work*,* and then there’s this pressure to make changes on top of it all.”* (I8).

Furthermore, insufficient support from significant others was perceived as a barrier to long-term commitment. Moreover, many individuals encountered health issues such as depression, anxiety, and other mental health challenges, along with physical ailments like pain, injuries, and loneliness, all of which hindered progress.

“*There’s no support from the family. You can really see the difference*,* if a friend or partner supports and gets involved in this*,* it’s really meaningful*,* but then you also see those cases where the family doesn’t support or doesn’t want to change their diet or increase physical activity together*,* and it’s been quite unpleasant*,* like if the partner drinks a lot of alcohol and doesn’t want the client change that.”* (I2).

Moreover, unsuccessful weight management can be attributed to various factors surrounding lifestyle habits in everyday life. For example, the coaches shared examples of individuals with indulgent habits, such as frequent dining out and alcohol consumption, making weight management challenging. Additionally, limited physical activity due to obesity or physical restrictions further complicated efforts.

“*I have a few who are morbidly large to the point where they can’t really move. It’s been interesting*,* you know*,* how one of them described it really well. Just thinking about these exercise services*,* even though they’re supposed to be accessible*,* but in reality*,* they don’t quite meet the needs. Like*,* you might not even be able to get into the pool*,* maybe because the steps are too difficult*,* sure*,* big swimming pools might have those ramps*,* but a smaller*,* private one mostly doesn’t. Or*,* for example*,* being that large that you can’t go to the beach because you sink so much in the sand.*” (I5).

Despite understanding weight management principles, many struggled to apply them effectively in their daily lives. Unhealthy dietary habits, including a reliance on energy-dense foods and a preference for fast food over vegetables, also impeded progress. The issues related to eating management played a significant role in hindering successful weight loss. For instance, the absence of a feeling of fullness and constant bottomless hunger complicated eating regulation. Additionally, eating disorders, particularly binge-eating disorder, where individuals use food for comfort or consolation, can significantly impede weight management efforts.

While most aspects of the Healthy Weight Coaching program were well-received, some areas were identified for improvement. Although standardized sessions benefited many patients, some desired a more flexible approach, especially those committed to continuity. The coaches noted areas where digital guidance could be enhanced to better support patients. They emphasized the importance of real-time communication and personalized interaction, suggesting that additional opportunities for dialogue and support throughout the program could further enhance the patient experience. Moreover, they recognized the value of intensive therapeutic approaches for those facing severe issues such as deep depression or trauma, and they actively encouraged patients to explore additional support options available through other service providers.

“*Many of my clients have had a very insecure childhood*,* really awful things happened*,* and those challenges related to food and eating management as well as weight gain have started since childhood*,* and there have been some really painful issues there. As I mentioned*,* many have undergone psychotherapy*,* but even that has been too little to address such huge issues*,* so it seems that probably quite a few have quite painful issues there that they’re not ready to deal with*,* and then it’s connected to eating.*” (I5).

Finally, the prevalent societal norms and cultural expectations regarding weight management in Western culture contributed to the challenges of lifestyle change. Unrealistic goals, as one of the coaches (I4) described: “*I asked if she would be satisfied with a smaller weight loss*,* and she responded that it* (referring to the unrealistic weight loss goal) *wasn’t her own wish*,* but rather she thought it was expected here*”, as well as pervasive dieting culture and intense pressure to achieve weight loss success are factors that influence patients’ thinking and behavior, often hindering progress and leading to disappointment and discontinuation of coaching.

*“The ‘all or nothing’ mentality can be a significant barrier. Whether it’s expecting everything to happen immediately or feeling like I have to change everything right away*,* neither approach is realistic. No one can sustain that kind of pressure; if they can*,* it’s only temporary. It leads to fatigue*,* and people often revert back to their old habits rather than attempting to stay on track.”* (I2).

*“Well*,* surely*,* a strong dieting background can be a hindrance for some if they haven’t really moved past the mindset of strict dieting or if they’re still heavily invested in it. In those cases*,* weight might drop*,* but then the methods they’re using to lose weight might not be sustainable in the long term*,* so that’s also a bit of a challenge.”* (I3).

## Discussion

The results of our study suggest significant positive changes in the patients participating in the Healthy Weight Coaching program, as noted by their coaches. These changes were evident across both psychological and behavioral domains, indicating improved strategies for weight management. Notably, the observed shift in the patients’ priorities emphasized holistic well-being over mere weight reduction, suggesting the program’s effectiveness in promoting a more balanced and sustainable approach to weight management.

However, the traditional success criteria in weight loss and lifestyle change studies have often prioritized quantifiable weight loss over well-being factors [8, 20]. Hall & Kahan [2] argued against focusing solely on weight loss as a success measure. Instead, they advocated for sustained improvements in diet quality and physical activity, which can contribute significantly to overall health outcomes, even without substantial weight loss.

Furthermore, Hall & Kahan [2] emphasized the significance of cognitive restructuring, which resonates with the present study’s findings. The coaches had observed a decline in negative self-perception and an increase in self-belief among the patients of the Healthy Weight Coaching program over time. Addressing and reshaping negative beliefs and maladaptive coping strategies, such as binge eating in response to minor setbacks, may alleviate behavioral fatigue and bolster resilience in the face of challenges [2]. This observation aligns with the results of a qualitative study investigating participants’ experiences in the 12-month Healthy Life Center program [21], where participants reported reduced catastrophic thinking and a shift from self-blame to self-compassion, consistent with the findings of the present study.

Hall & Kahan [2] also underscored cognitive flexibility, noting that rigid, all-or-nothing approaches can hinder long-term success. They proposed abandoning unrealistic weight and lifestyle goals to foster adaptability and resilience. This observation resonates with findings from the current study, where the coaches noted an increase in patients’ flexibility and moderation in their eating habits. This perspective aligns with previous research indicating that the individuals with huge and unrealistic weight loss expectations may face adverse outcomes, including dissatisfaction and learned helplessness [22, 23]. Furthermore, the healthcare professionals may also harbor unrealistic expectations regarding weight loss outcomes [24], reflecting broader societal attitudes toward weight and weight management. These cultural norms were evident among the Healthy Weight Coaching program patients, who initially prioritized weight-related progress at the program’s outset.

Moreover, prioritizing well-being aligns with research indicating that even modest weight loss improves cardiovascular health [25]. Therefore, a comprehensive approach like that offered in the Healthy Weight Coaching program supports sustainable lifestyle changes and counters unrealistic expectations.

The observed decrease in binge eating behaviors during the Healthy Weight Coaching program aligns with the broader body of research indicating that structured weight loss interventions can positively impact eating behaviors. Several studies support that clinical interventions for weight loss, such as dietary interventions and behavior management programs, can contribute to decreased severity and frequency of binge eating, particularly during treatment [26, 27]. However, a recent review reported that individuals with and without binge eating tendencies did not differ in weight loss outcomes during weight loss treatment [28]. This suggests that individuals who continue to exhibit binge eating patterns may still achieve success in their weight loss efforts.

The positive behavioral changes observed in the present study are consistent with previous research on lifestyle interventions improving health outcomes. For example, Smith et al. [29] reported eating pattern improvements with dietary counseling, while Papadopoulos et al. [30] highlighted adequate sleep, and Carraça et al. [31] reported the importance of enjoyment in physical activity. Moreover, Jessen-Winge et al. [32], in their qualitative investigation into the preferences of individuals with obesity regarding weight loss programs, found that emphasizing the gradual development of new habits and routines integrated into daily life was crucial for program efficacy.

We also found several factors that facilitated success in the Healthy Weight Coaching. Personal factors such as motivation and high self-efficacy emerged as crucial determinants in the patients’ ability to achieve their weight management goals and sustain lifestyle changes. These findings are consistent with those of a qualitative review by Tay et al. [33], who reported that higher self-efficacy predicted proactive weight management behaviors, such as modifying the food environment, adopting healthier habits, and seeking support when needed. This pivotal role of high self-efficacy in successful weight management is further supported by several other studies [20, 34, 35].

Furthermore, Tay et al. [33] observed that achieving weight loss milestones supported participants’ motivation to persist in their efforts. Conversely, unmet weight loss expectations were found to diminish motivation to continue with the intervention. While external motivators, such as participation in the coaching program and the desire to demonstrate competence to others, initially facilitated behavior change, our study, in line with the qualitative review by Tay et al. [33], recognized that these may not be sufficient for long-term maintenance. In another systematic review of qualitative studies exploring the barriers and facilitators of digital health interventions, the authors also underscored the pivotal role of internal motivation in managing healthy lifestyle changes [36], as was similarly highlighted in a mixed-methods study investigating the interaction between primary care physicians and women who had achieved weight loss [37].

Sevild et al. [21] also echoed this observation, highlighting that health behavior changes motivated by external pressures from sources like family, doctors, or societal standards often encounter challenges in sustainability. Conversely, changes driven by personally significant values and preferences tended to endure. Additionally, internal motivation has consistently been associated with successful lifestyle changes and long-term weight maintenance in quantitative studies as well [38–40].

Furthermore, features of the digital Healthy Weight Coaching program, such as its personalized approach and accessibility, were seen as significant in supporting lifestyle change. This aligns with the findings from a qualitative study among men of low socioeconomic status, which emphasized the need for weight loss programs to adapt to the participants’ lives, stressing the importance of integrating dietary changes into daily routines and allowing for personalization [41]. Similarly, a tailored approach was appreciated by newly-becoming mothers with obesity, who found diet and activity advice particularly useful when tailored to their personal challenges and fitting into their lives [42]. Accessibility was universally beneficial, allowing participants to engage conveniently from anywhere, including their homes, which aligns with the findings that treatment availability -more than distance- was key [43]. Prolonged waiting times for treatment were linked to discontinuation of interventions.

The integral role of the coaches emerged as a cornerstone of the Healthy Weight Coaching program. They were instrumental in tailoring the coaching experience to meet each patient’s unique needs, providing consistent support throughout their journey. Furthermore, the patients valued having a consistent support system throughout their journey towards the lifestyle change. Various studies have shown that strong relationships with the healthcare providers are crucial in both digital and in-person programs [8, 21, 36, 41, 44–47]. Tay et al. [33] highlighted in their review the crucial role of personalized support and accountability in intervention success, emphasizing the therapeutic relationship between the participants and the healthcare professionals. Similarly, Jessen-Winge et al. [32] found in their interview study that the participants valued the health professionals’ knowledge-sharing and encouragement throughout their weight loss journey. Banerjee et al. [37] also noted the positive impact of celebrating small successes on sustained motivation: the participants were more likely to continue their weight loss efforts when the healthcare professionals recognized small weight losses and positive behavior changes, providing comments and encouragement. Consistent with our findings, wherein the coaches shared examples of the patients expressing gratitude for their support and encouraging words. Moreover, research conducted on both digital and face-to-face lifestyle change programs underscores the importance of family and peer support in achieving success in weight loss efforts [8, 33, 44, 46, 48]. These findings underscore the importance of personal support and human interaction in weight management interventions, even when delivered through digital platforms.

Despite the observed positive outcomes, participants in the Healthy Weight Coaching program encountered various barriers, such as challenging life circumstances, mental health issues, and limited social support that hindered their progress in weight management. These findings are consistent with previous research. For instance, a systematic review highlighted a strong association between adverse life experiences and obesity development [49]. Moreover, challenging life situations have been linked to higher drop-out rates in weight reduction programs [50] as well as mental health challenges that complicate maintaining weight practices [51]. Notably, good mental health has been associated with program completion [43]. Furthermore, a systematic review of qualitative studies recognized the role of challenging life circumstances and everyday obstacles in undermining weight management efforts [52].

The qualitative research by Norman et al. [53] illuminated the intricacies of eating behavior and weight management, highlighting life circumstances, anxiety, and mental health issues as significant barriers. Similarly, insights from Funk et al. [54], focusing on bariatric surgery patients, unveiled additional challenges such as long working hours and difficult life circumstances that hinder weight management efforts, exacerbated by income-related difficulties. Further exploration by Norman et al. [55] delved into the experiences of individuals with lower socioeconomic status, revealing similar hurdles in weight management: limited financial resources compelled participants to carefully consider their actions, posing challenges in affording participation in physical activities or maintaining a healthy diet.

Finally, societal norms and expectations surrounding weight management present challenges to the success of the Healthy Weight Coaching patients. In contemporary Western society, the pursuit of a healthy weight has become deeply ingrained in cultural norms and societal expectations [56]. The pressure for individuals to conform to narrow physical appearance standards often propels a relentless pursuit of weight management goals, as depicted by the coaches’ narratives of their patients’ experiences. Similar sentiments were echoed by newly-becoming mothers with obesity, who expressed feelings of ‘naughtiness’, ‘embarrassment’, or ‘shame’ due to their weight, particularly in interactions with healthcare professionals [42]. However, within specialized clinics catering to individuals with obesity, they reported feeling appreciated and free from stigma.

Weight related pressures are internalized from an early age, as shown by Davis et al. [57], who found that social expectations, especially for white women, emphasized thinness from childhood, with overweight viewed as unacceptable. Additionally, another qualitative study highlighted negative experiences with healthcare professionals in managing obesity. These professionals were perceived as unhelpful and contributing to stigma, with their language exacerbating feelings of upset and defiance among participants. Furthermore, participants felt dismissed by the healthcare professionals who solely attributed weight gain to diet without considering other factors [58].

### Strengths and limitations

The findings of this study should be contextualized within its methodological strengths and limitations. The qualitative interview studies offer a robust methodology for delving into the perspectives, experiences, and attitudes of individuals. This approach provides flexibility, enabling researchers to adapt questions to interviewee responses and unearth unexpected insights. Moreover, active engagement and collaboration between the researcher and interviewees contribute to richer and more nuanced research findings, thereby enhancing the study’s validity.

One notable strength of this study was the careful selection of healthcare professionals, specifically coaches, owing to their integral role in the digital obesity treatment program. Additionally, maintaining consistency was ensured by employing the same interviewer throughout the study, guaranteeing uniformity in interview conduct across all the participants.

However, it’s important to acknowledge the limitations of this study. Like all qualitative studies, researcher bias may emerge from preconceptions, experiences, and perspectives. Active discussion and reflection on study findings occurred during analysis to mitigate this. Additionally, there is a potential risk of social desirability bias in interview responses [59], especially when participants are from the same work team evaluating their own program. However, the interviewer’s role as a researcher differs significantly from that of the coaches, and while there is a shared background in obesity and weight management, the interviewer’s limited direct engagement with the coaches provided a fresh perspective during the interviews. Prior research literature also informed the themes discussed, ensuring that important issues were thoroughly explored. The coaches openly shared their thoughts, trusting in the interviewer’s assurance of anonymity.

As digital interventions are increasingly used in weight management programs, they also bring challenges related to increased screen time and potential sedentary behavior. While smartphones and other digital devices offer convenient access to health interventions, their use has been linked to sedentary lifestyles and related health concerns [6, 7]. To address these risks, the Healthy Weight Coaching program incorporates features that reduce passive screen time. For example, participants can listen to weekly session summaries via podcasts, allowing them to engage with the content while walking or performing other activities. Additionally, movement-focused sessions promote physical activity during the program. The program also encourages regular breaks from sitting still, reminding participants to interrupt prolonged periods of inactivity.

Moreover, the program may serve as a positive alternative to excessive social media use. Patients are encouraged to connect with their coach and fellow patients for peer support, offering a meaningful, health-oriented interaction that can fulfill the social needs often met by social media browsing. These adaptations ensure that the digital format promotes healthier habits and social connection, rather than exacerbating sedentary behavior or contributing to unhealthy screen time.

Furthermore, while our study did not specifically focus on change resistance, this aspect presents an interesting avenue for future research. As our research employed a data-driven thematic analysis to explore success factors in a digital lifestyle intervention, we concentrated on the themes that emerged directly from the data. However, incorporating a theoretical framework, such as Colin Carnall’s Coping Cycle Model [60], which examines how individuals respond to and cope with change, could have provided additional insights into how patients resist or adapt to the changes required by the program.

While our findings highlight key barriers such as burdensome life situations, limited resources, and psychological challenges, future research could benefit from examining change resistance more explicitly. Integrating such frameworks might offer a deeper understanding of the patients’ behavioral and psychological processes, potentially helping to tailor interventions more effectively to support individuals through the various stages of change.

Finally, it is important to acknowledge that the study’s findings are derived from the coaches’ perspectives and their interpretation of the patients’ experiences in the Healthy Weight Coaching program. Interviewing the patients may yield different themes and shed light on alternative aspects of the program’s effectiveness. Additionally, examining the successes and roles of the coaches in the coaching process in greater depth could provide valuable insights and offer important information on how digital coaching programs could be further developed and optimized. Understanding how the coaches’ experiences and reflections influence both the patients and the outcomes could be a key factor in improving the effectiveness of such programs.

## Conclusion

The findings of this study hold significant implications for refining weight management interventions. By acknowledging individual needs and providing tailored support, digital interventions can enhance their effectiveness and foster sustained success. This entails a multifaceted approach, including personalized adjustments, improved accessibility, and challenging societal norms surrounding weight management.

In essence, this qualitative inquiry illuminates the triumphs and challenges encountered within the digital Healthy Weight Coaching program, offering crucial insights into the factors shaping success. It underscores the pivotal role of personalized support, trust, and rapport between the coaches and the patients in driving positive outcomes. While the program excels in fostering a supportive environment, opportunities for enhancement lie in addressing communication barriers and ensuring timely responses to patient inquiries.

Looking ahead, future research should prioritize evaluating the long-term effectiveness and scalability of digital weight management programs. Innovative strategies to bolster the participant engagement and adherence also warrant exploration. By continuously refining interventions to meet the diverse needs of the participants, we can advance the development of more effective and accessible approaches to promoting healthy lifestyle changes and weight management.

## Supplementary Information

Supplementary Material 1.

## Data Availability

Due to confidentiality agreements and to protect the privacy of the study participants, the data collected for this research cannot be shared openly. We made assurances to our informants that their data would be kept confidential and not shared beyond the scope of this study. We apologize for any inconvenience this may cause and are committed to upholding the privacy and confidentiality of our participants.
