# Supplementary material for: Exploring successes, barriers, and enablers in the one-year digital Healthy Weight Coaching
Source: BMC Health Serv Res. 2024 Nov 8;24:1367. doi: 10.1186/s12913-024-11876-2 (PMC11549835; doi:10.1186/s12913-024-11876-2)
Supplement: Supplementary file 1 — Supplementary Material 1. [file 12913_2024_11876_MOESM1_ESM.docx]

Interview guide

Background Information:

Age, gender, education?

Coaching experience?

Number of clients in the Healthy Weight Coaching?

1. What do you consider success at the Healthy Weight Coaching?

- What factors do you think can generally explain a successful outcome?
- What factors do you think can explain a so-called unsuccessful outcome?
- How have patients justified their decision to discontinue the Healthy Weight Coaching?
- Based on your coaching experience, can you identify any predictive factors for optimal/non-optimal progress in coaching?

2. What factors assist the lifestyle change in the Healthy Weight Coaching?

- What is the role of the coaches?
- What is the role of the patients?
- What is the role of the program?
- What are the challenges?

3. The Healthy Weight Coaching program - why does it work?

- Who do you think this coaching is suitable for?
- What has worked particularly well on the path?
- How would you improve coaching?

Additional remarks
